# Microbial Community Succession and Its Correlation with Quality Characteristics during Gray Sufu Fermentation

**DOI:** 10.3390/foods12142767

**Published:** 2023-07-20

**Authors:** Lei Zhao, Yang Liu, Qiong Xu, Yi Yu, Guojian Zheng, Yue Wang, Qingping Zhang, Xiaoqian Xu, Nana Zhang, Jiayue Chu, Yuzhu Zhang, Yingyi Sun, Qin Zhao, Yinan Zhang, Qinfeng Qu, Jiang Zhong

**Affiliations:** 1 Key Laboratory of Milk and Dairy Products Detection and Monitoring Technology for State Market Regulation, Shanghai Institute of Quality Inspection and Technical Research, Shanghai 200233, China; zhaolei19890117@163.com (L.Z.); xuqiong@sqi.org.cn (Q.X.); yuyi@sqi.org.cn (Y.Y.); zhenggj@sqi.org.cn (G.Z.); wangyue@sqi.org.cn (Y.W.); zhangqp@sqi.org.cn (Q.Z.); xuxq@sqi.org.cn (X.X.); zhangnn@sqi.org.cn (N.Z.); zhujy@sqi.org.cn (J.C.); zhangyz1@sqi.org.cn (Y.Z.); sunyy@sqi.org.cn (Y.S.); zhaoqin@sqi.org.cn (Q.Z.); zhangyn@sqi.org.cn (Y.Z.); quqf@sqi.org.cn (Q.Q.); 2 Department of Microbiology and Microbial Engineering, School of Life Sciences, Fudan University, Shanghai 200438, China; jzhong@fudan.edu.cn

**Keywords:** fermented food, sufu, microbial diversity, biogenic amine, correlation analysis

## Abstract

Gray sufu, a traditional fermented food derived from soybeans, undergoes a complex fermentation process. This study aimed to investigate the dynamics of the microbial community during sufu fermentation and its relationship with key quality characteristics. Through systematic sampling of sufu at different phases of fermentation, 143 bacterial genera and 84 fungal genera involved in the process were identified. Among these, *Chishuiella*, *Enterococcus*, *Lactococcus*, and *Weissella* emerged as the predominant bacterial communities. After seven days of ripening fermentation, *Trichosporon* supplanted *Diutina* as the predominant fungus, accounting for more than 84% of all fungi. Using redundancy analysis, significant correlations between microbiota and physicochemical properties were uncovered. *Chishuiella* and *Empedobacter* displayed positive relationships with pH, soluble protein, and amino nitrogen content. In addition, five biogenic amines were detected, and it was determined that tyramine accounted for more than 75% of the total biogenic amines in the final gray sufu products. Spearman correlation analysis revealed significant positive relationships between *Lactococcus*, *Enterococcus*, *Tetragenococcus*, *Halanaerobium*, and *Trichosporon* and the five biogenic amines examined. These findings shed light on the complex interactions between microorganisms and biogenic amines during the fermentation of gray sufu, thereby facilitating the development of microbial regulation strategies for better quality control.

## 1. Introduction

**Figure 1:**
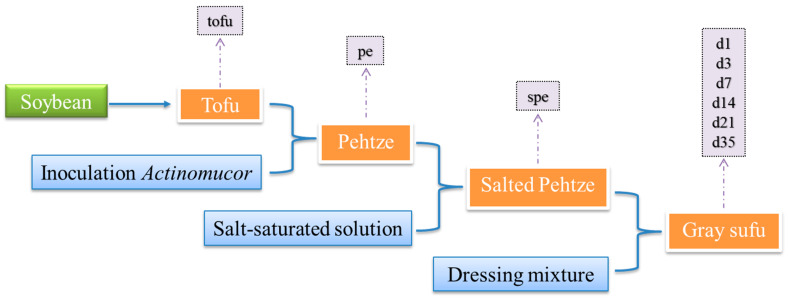
Production process and sampling points for gray sufu. Pehtze (pe) was produced by inoculating tofu (tofu) with *Actinomucor* for 24 h and then salting for 2 days to achieve salted pehtze (spe). Then, the salted pehtze was ripened for 1 (d1), 3 (d3), 7 (d7), 14 (d14), 21 (d21), and 35 (d35) days.

Fermented foods have been an integral part of human diets for millennia, offering numerous health benefits and enhancing the sensory characteristics of food [1]. A wide variety of foods, such as cereals, milk, fish, and other meats, soybeans, fruits, and vegetables, have been fermented using traditional components [2]. Sufu, a fermented soybean product, has gained popularity in the East as a condiment and dietary supplement [3]. Depending on the types of starter introduced, sufu can be classified into three types: mold-fermented sufu (inoculated with *Actinomucor*, *Mucor*, or *Rhizopus*), bacteria-fermented sufu (inoculated with *Bacillus* or *Micrococcus*), and others (naturally inoculated) [4]. Among all sufu types, mold-fermented sufu is the most prevalent [5]. Gray sufu, which has a distinctive aroma and a delightful flavor, is a typical mold-fermented sufu in China. It is receiving attention for its potential health benefits, especially its high levels of the bioavailable ingredient S-equol (4′, 7-dihydroxy-isoflavandiol), which may contribute to preventing bone loss and reducing menopausal symptoms [6]. In addition, gray sufu is a rich source of vitamin B_12_, making it a valuable supplement for a vegan diet [7]. The processing of gray sufu can be approximately divided into four phases (Figure 1). Firstly, tofu cubes are created by salt precipitation of the boiling soymilk. Starter fungi are then introduced to the tofu cubes, which are incubated until fungal hyphae cover them, forming moldy tofu. The moldy tofu cubes are pickled in salt for several days after removing the mycelium from their surfaces. Afterward, the salt-cured moldy tofu blocks are canned and aged for one to two months in a sealed container with the dressing mixture [8].

The microbial community composition in gray sufu varies significantly due to geographical features and manufacturing environments, as it is produced through open fermentation [9]. In Changchun province of Northeastern China, the core microbial genera identified in gray sufu fermentation were *Lactobacillus*, *Bacillus*, *Pediococcus*, *Pseudomonas*, *Apiotrichum*, *Sarocladium*, and *Trichosporon* [5]. In Hebei province of North China, *Fusobacterium* and *Providencia* were found to be the main microbial genera involved in gray sufu fermentation [10]. However, the microbial communities and their changes during gray sufu fermentation in other regions remain largely unknown. Understanding the dynamic changes and microbial sources in gray sufu fermentation is essential for efficient quality and safety management.

Microorganisms have a significant impact on the physicochemical and safety properties of gray sufu [11]. Various nutritional indicators, including amino nitrogen and soluble protein content, are closely related to the maturity of gray sufu, while pH and NaCl levels are indicators of its quality. Due to its complex microbial structure, gray sufu’s quality can be inconsistent and may pose food safety risks. Biogenic amines (BAs), produced by decarboxylase-producing microorganisms in foods, can pose toxicological risks when consumed in excessive amounts [12]. Detection of BAs in food is, therefore, of great interest due to their potential adverse effects, such as hypertension, vomiting, perspiration, and headaches [13,14]. Regulatory guidelines recommend that the total biogenic amine content in food should not exceed 900 mg/kg [15], with histamine levels not exceeding the legal limit set by the Food and Drug Administration (FDA) at 50 mg/kg [16]. Gray sufu, a traditional fermented food with a high amino acid content, may provide a suitable environment for the production of BAs [17]. Monitoring the dynamics of BA distribution and content at different phases of gray sufu production is necessary to elucidate the key technological points associated with BA production.

Shaoxing gray sufu, a mold-fermented product inoculated with *Actinomucor*, holds a significant market share in eastern China and is gaining popularity as an export. To gain a deeper understanding of the complex fermentation process of Shaoxing gray sufu, a comprehensive study was conducted to investigate the diversity and succession patterns of microbial communities, analyze variations in physicochemical properties, BAs, and amino acid content, and establish correlations between the microbiota and the physicochemical properties and BAs. These results provide a solid foundation for monitoring microbial development and tracing product quality, ultimately enhancing the safety and overall quality of gray sufu.

## 2. Materials and Methods

### 2.1. Sample Collection

In this study, samples were collected from Shaoxing, Zhejiang Province, China. As depicted in Figure 1, pehtze was produced by inoculating tofu cubes with *Actinomucor*, salting the pehtze for 2 days to produce salted pehtze, then dispersing the salted pehtze into wide-mouthed glass vessels and ripening with the dressing mixture at 25 °C for 35 days. At each phase, three samples were collected to evaluate microbial diversity, physicochemical indicators, biogenic amine content, and amino acid content. The samples were stored at −20 °C.

### 2.2. Extraction of Nucleic Acids

The tofu cubes (15 g) were crushed and vortexed with 30 mL of sterile water in a sterile bag, and the mixture was incubated at room temperature for 10 min. Then, 10 mL of the sample solution was centrifuged at 3000× *g* for 5 min. Subsequently, the supernatant was transferred to a new tube and centrifuged at 10,000× *g* for 5 min to collect the precipitate. After washing the precipitate twice with 5 mL of sterile water, nucleic acid extraction was performed using the CTAB method [18]. The extracted DNA samples were eluted in 100 µL of TE buffer (prepared from 1M stock Tris-Cl (pH 7.5) and 500 mM stock EDTA (pH 8.0)), and their quality was assessed by measuring the OD_260_/OD_280_ and OD_260_/OD_230_ ratios with a Nanodrop 2000 Spectrophotometer (Thermo Fisher Scientific, Wilmington, MA, USA) [19]. DNA quantification was performed using a Qubit 3.0 Fluorometer (Life Technologies, Carlsbad, CA, USA) and a dsDNA HS Assay Kit (Life Technologies, Carlsbad, CA, USA) [20]. Quantification was performed using the included DNA standards of 0 ng/μL and 10 ng/μL according to the manufacturer’s instructions. The samples were stored at −20 °C.

### 2.3. PCR Amplification and Sequencing

The bacterial 16S rRNA gene sequences were amplified with primers 338F (5′-ACTCCTACGGGAGGCAGCAG-3′) and 806R (5′-GGACTACHVGGGTWTCTAAT-3′) [21]. For the fungal ITS region, primers ITS1F (5′-CTTGGTCATTTAGAGGAAGTAA-3′) and ITS2R (5′-GCTGCGTTCTTCATCGATGC-3′) were used [22]. The 20 μL PCR amplification system consisted of 4 μL of 5×FastPfu buffer, 2 μL of dNTPs (2.5 mM), 0.8 μL of forward primer (5 μM), 0.8 μL of reverse primer (5 μM), 0.4 μL of FastPfu polymerase, 0.2 μL of BSA, 10 ng of template DNA, and deionized water. PCR amplification was carried out on an ABI GeneAmp^®^ 9700 (Applied Biosystems, Waltham, MA, USA) with the following conditions: initial denaturation at 95 °C for 3 min, 35 cycles of 95 °C for 30 s, 55 °C for 30 s, 72 °C for 45 s, and a final extension at 72 °C for 10 min [23]. The amplified products were purified using the AxyPrep DNA Gel Extraction Kit (AXYGEN Biosciences, Union City, CA, USA) [24] and then sequenced using the Illumina MiSeq platform by Major Bio (Shanghai, China).

The data were analyzed using the Major Bio i-Sanger Cloud Platform (www.i-sanger.com, accessed from 15–30 March 2023) [25]. Details of the analysis are described below. The raw data underwent quality filtration using Fastp software (version 0.19.6, https://github.com/OpenGene/fastp, accessed on 15 March 2023) to remove low-quality sequences [26]. Operational taxonomic units (OTUs) were obtained by dereplicating, removing singletons, and deblocking chimeric sequences using Usearch software (version 7.0, http://www.drive5.com/usearch/, accessed on 24 March 2023) with a 97% similarity clustering threshold [27]. For bacterial taxonomy assignment, the Silva database (Release 138, http://www.arb-silva.de, accessed on 25 March 2023) was employed [28], and for fungal taxonomy assignment, the Unite database (Release 8.0, http://unite.ut.ee/index.php, accessed on 26 March 2023) was used [29]. Taxonomic assignment was performed using the RDP classifier implemented in QIIME software (version 1.9.1, http://qiime.org/scripts/assign_taxonomy.html, accessed on 30 March 2023) [30]. To improve the accuracy of tracking microbial community evolution during sufu fermentation, the following screening criteria were applied to the OTUs: removal of OTUs that matched chloroplast and mitochondrial sequences and determination of parity based on the minimum sample sequence number [31,32].

### 2.4. Physicochemical Analysis

The pH levels of the samples were determined in accordance with the GB 5009.237–2016 National Standard for Food Safety. A total of 2 g of the sample was transferred to a 50 mL beaker, followed by the addition of 18 mL of pre-boiled and cooled distilled water. The mixture was then stirred to ensure homogeneity, and the pH of the solution was measured at 20 °C using a pH meter (FE28-m, Mettler Toledo, Schwerzenbach, Switzerland) [33].

The salt content of the sample was measured in compliance with GB 5009.44-2016 National Standard for Food Safety by following a method that involved the addition of a 10 g of sample to 50 mL of 70 °C water, sonication for 20 min, cooling to room temperature, and filtration. Then, an equal volume of water was added to 50 mL of the filtrate, and the solution was titrated with AgNO_3_ (1 M) using 10% (*w*/*v*) K_2_CrO_4_ (1 mL) as an indicator. The volume of titrant required to change the color of the solution from yellow to orange was then recorded.

The evaluation of moisture content, total acid, amino nitrogen, and soluble protein content in the samples followed the PRC Domestic Trade Industry Standard SB/T 10170-2007 with minor modifications. To determine the moisture content of the samples, 5 g of the samples was dried continuously at 105 °C. The difference in mass before and after drying was used to calculate the moisture content. Total acid and amino nitrogen content were determined by mixing 20 g of sample with 80 mL of 60 °C water, boiling, cooling, and filtering. An automated potentiometric titrator was used to determine the amount of NaOH (0.05 M) used for titration. Filtrate (10 mL) was combined with 50 mL of water, and the titration was conducted at pH 8.2 for total acid calculation and at pH 9.0 for amino nitrogen calculation. For the soluble protein assay, the pretreatment was similar to that of the total acid samples. Filtrate (10 mL) was combined with 15 mL of HCl (1 M) and digestion tablets in a digestion tube. After achieving a green and transparent liquid in the digestion tube, the solution was allowed to cool. The resulting solution was distilled and titrated using a fully automated protein tester.

### 2.5. Biogenic Amines and Amino Acids Analysis

To analyze BA content, the method proposed by Ochi et al. was adapted as follows [34]. A 1 g sample was mixed with 10 mL of extraction solution (acetonitrile:water:formic acid = 70:29:1), followed by vortexing for dispersion and sonication for 30 min. After centrifugation at 8000× g for 2 min, the supernatant was filtered through a 0.22 μm PTFE membrane to obtain the sample solution. The BA content was determined using liquid chromatography-tandem mass spectrometry (LC-MS/MS) methods. Liquid chromatography was performed using an Agilent Prooshell PFP (4.6 mm × 100 mm, 4 μm) column with gradient elution of mobile phase A (0.1% formic acid aqueous solution) and mobile phase B (acetonitrile). During the 11-min run, the percentage of mobile phase A varied as follows: 0–1 min, 95% A; 1–6 min, linear decrease in A from 95% to 0%; 6–8 min, 0% A; 8–11 min, increase in A to 95%, followed by 2 min of equilibration. The injection volume was set to 2 μL and the injection flow rate to 0.3 mL/min. For mass spectrometry analysis, positive ionization mode with an electrospray ionization (ESI) source was employed, utilizing the following parameters: capillary voltage of 3.0 kV, cone well voltage of 30 V, drying gas temperature of 400 °C, drying gas flow rate of 800 L/h, cone well blowback gas flow rate of 150 L/h, and ion source temperature of 150 °C. Specific information on parameters such as ion pairs, collision energies, and elution time of each biogenic amine was presented in Supplementary Table S1. Histamine, putrescine, and tyramine standard products were purchased from Shanghai Yuanye Biotechnology Co., Ltd. (Shanghai, China). Phenethylamine and cadaverine standard products were obtained from Tmstandard Company (Beijing, China).

To investigate the relationship between biogenic amine synthesis and amino acid metabolism, the amino acid content of each sample was determined according to the GB 5009.124-2016 National Standard for Food Safety. A hydrolysis tube containing 1 g of sufu sample and 10 mL of 6 mol/L hydrochloric acid solution underwent a freezing step for 5 min, followed by evacuation and placement at a controlled temperature of 110 ± 1 °C for hydrolysis. The hydrolysate was filtered and adjusted to a final volume of 50 mL. A 1.0 mL aliquot of the filtrate was then subjected to reduced-pressure drying, followed by reconstitution in an equal volume of sodium citrate buffer solution (pH 2.2). The resulting solution was further filtered through a 0.22 μm membrane to obtain the sample solution. Subsequently, 20 µL of the sample solution was injected into a high-speed amino acid analyzer (High-speed Amino Acid Analyzer L-8900, Hitachi High-Technologies Corporation, Tokyo, Japan) equipped with a Hitachi custom ion exchange column (Hitachi High-Technologies Corporation, Tokyo, Japan). Method details are described in Supplementary Table S2. The results were expressed as mg/100 g sufu, referring to an amino acid mixture standard (First Standard^®^, Alta Scientific, Tianjin, China).

### 2.6. Statistical Analysis

Each experiment was performed thrice. The results were subjected to one-way ANOVA analysis and Tukey’s multiple comparisons tests using Graphpad Prism (version 6.0), with *p* < 0.05 considered statistically significant. Redundancy analysis (RDA) was applied to assess the relationship between abundant microorganisms (OUT > 1%) and physicochemical properties using Canoco (version 5.0, http://www.canoco5.com/, accessed on 6 April 2023). Spearman’s correlation coefficient was used to evaluate the correlations between the 50 most abundant microbial taxa and BAs. A heatmap was generated using pheatmap R packages (version 3.3.1).

## 3. Results

### 3.1. Dynamics of Bacterial Community

**Figure 2:**
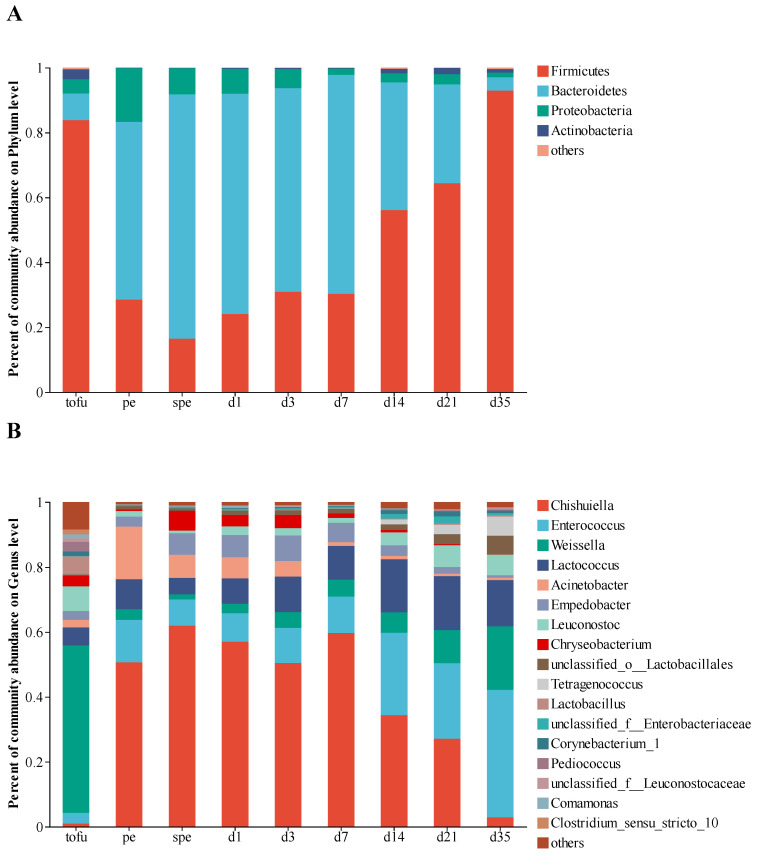
Bacterial composition in the gray sufu samples during fermentation. Data portray (**A**) phylum and (**B**) genus level of 16S rRNA gene sequences. For each sample, three repeats were performed, and the mean relative abundance was calculated. Phylum or genus with an abundance percentage of less than 1% were grouped together.

After quality control and screening, 27,640 bacterial sequences per sample were obtained, and 303 bacterial OTUs were identified. A phylum and genus level classification of bacterial 16S rRNA gene sequences was carried out in order to find out what makes up the bacterial community in gray sufu. In total, 13 phyla and 143 genera were identified. It was found that Firmicutes, Bacteroidetes, Proteobacteria, and Actinobacteria exhibited a relative abundance greater than 1% (Figure 2A). Among the 143 genera, the relative abundance of 17 genera all exceeded 1% (Figure 2B). In tofu, *Weissella* was the dominant genus. *Chishuiella* (from 0.99% to 50.71%), as well as *Acinetobacter* (from 2.31% to 16.18%) and *Enterococcus* (from 3.28% to 13.05%), increased rapidly in pehtze. *Chishuiella* reached its maximum relative abundance of 61.99% after salting. It was found that *Chishuiella*, *Enterococcus*, *Lactococcus*, and *Weissella* accounted for more than 76% of the relative abundance during the fermentation process. However, the relative abundance of *Chishuiella* decreased to approximately 3% after 35 days of ripening fermentation. As fermentation progressed, the relative abundance of *Enterococcus* increased, and that of *Lactococcus* and *Weissella* fluctuated. In addition, *Acinetobacter* abundance decreased continuously during the ripening process.

### 3.2. Dynamics of Fungal Community

**Figure 3:**
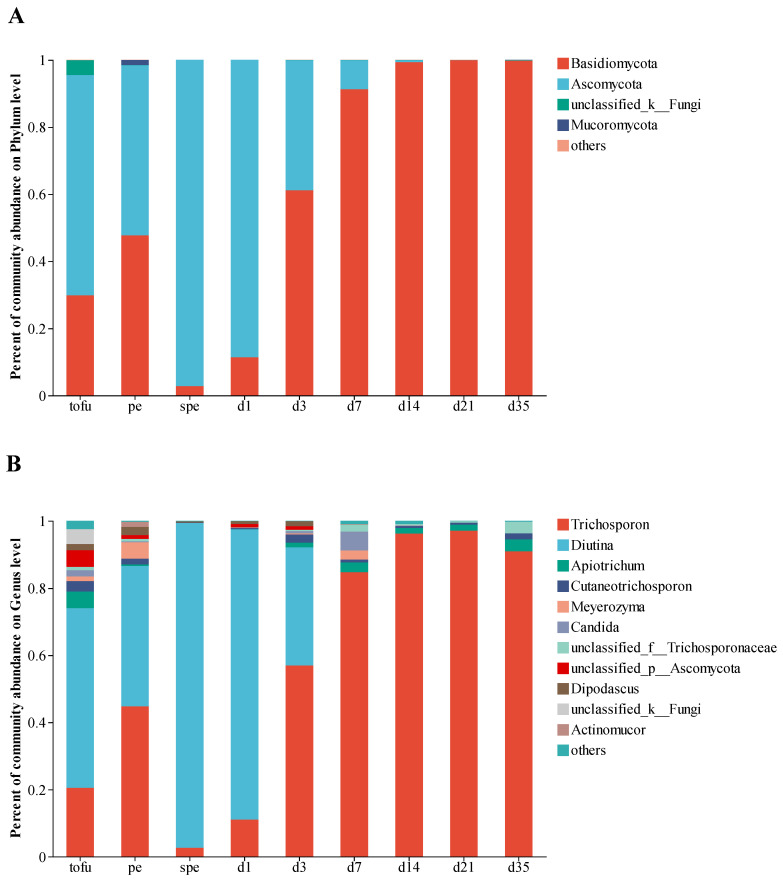
Fungal composition in the gray sufu samples during fermentation. Data portray (**A**) phylum and (**B**) genus level of ITS gene sequences. For each sample, three repeats were performed, and the mean relative abundance was calculated. Phylum or genus with an abundance percentage of less than 1% were grouped together.

A total of 31,928 fungal sequences were obtained per sample after quality control and screening, and 197 fungal OTUs were identified. The ITS gene sequences of fungi were classified at the phylum and genus levels in order to understand the dynamics of the fungal community during the fermentation of gray sufu. The gray sufu contained a total of 5 phyla and 84 genera. Four phyla, such as Basidiomycota, Ascomycota, and Mucoromycota (Figure 3A), exhibited relative abundances greater than 1%. Eleven of the eighty-four detected genera possessed relative abundances above 1%. The results showed that tofu samples had a sizable proportion of *Diutina* (53.54%). In pehtze samples, *Trichospron* (44.78%) and *Diutina* (41.87%) were the predominant genera. In salted pehtze samples, the proportion of *Diutina* was 96.75%. After 3 days of ripening fermentation, a significant shift in the dominant fungal genus was observed in our study. *Trichospron* replaced *Diutina* as the prevailing fungus, constituting over 84% of all fungi after 7 days of ripening fermentation (Figure 3B).

### 3.3. Physicochemical Properties during Sufu Fermentation

**Figure 4:**
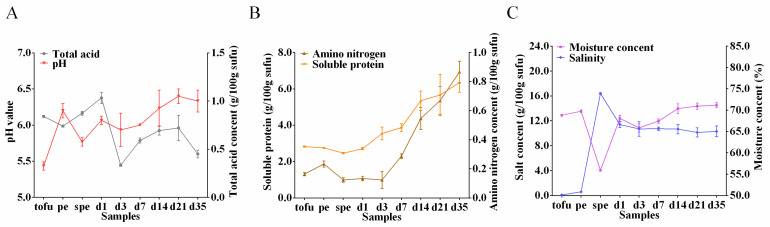
The alterations in total acid and pH (**A**), amino nitrogen and soluble protein (**B**), as well as moisture content and salinity (**C**) during sufu fermentation. The data are presented as means ± SD (*n* = 3). pe: pehtze; spe: salted pehtze; d1, d3, d7, d14, d21, and d35: the ripening time of sufu samples.

The variations in pH, total acid, moisture, salinity, amino nitrogen, and soluble protein during sufu fermentation are depicted in Figure 4. The original pH of tofu was 5.43, which climbed to 6.20 after inoculation, reduced to 5.77 during salted pehtze, and then raised to 6.33 towards the end of fermentation. The total acid content of tofu was 0.84 g/100 g, which decreased to 0.33 g/100 g after 3 days of ripening fermentation. As fermentation progressed, the total acid content tended to first increase and then decrease. During fermentation, proteases produced by microorganisms hydrolyze proteins [35], resulting in an increase in the levels of amino nitrogen and soluble protein to 0.87 g/100 g and 6.35 g/100 g, respectively. The salinity of tofu and pehtze was minimal, but it increased to 16.40 g/100 g in salted pehtze and remained over 10 g/100 g until the end of fermentation. The moisture content and salinity showed approximately opposing tendencies.

### 3.4. Biogenic Amine Production and Amino Acid Content during Sufu Fermentation

**Figure 5:**
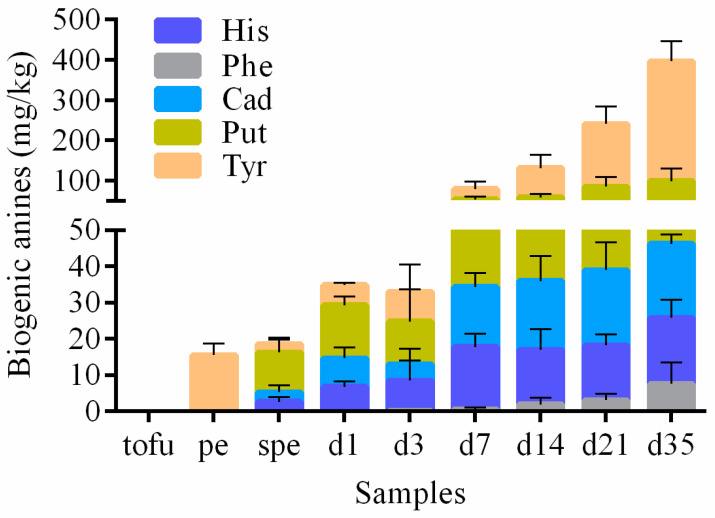
The variations in histamine (His), phenylethylamine (Phe), cadaverine (Cad), putrescine (Put), and tyramine (Tyr) during the sufu fermentation. pe: pehtze; spe: salted pehtze; d1, d3, d7, d14, d21, and d35: the ripening time of sufu samples.

During the production of sufu, five BAs were detected, and the concentration of these BAs changed, as shown in Figure 5. After 35 days of ripening fermentation, the total biogenic amine content reached 396.80 mg/kg. In this study, tyramine and putrescine were identified as the primary BAs generated during the production of gray sufu. At the end of fermentation, the content of tyramine reached 297.78 mg/kg, which was much higher than the content of other BAs. The production of putrescine, histamine, and cadaverine began in salted pehtze and increased as fermentation advanced. As shown in Figure 5, putrescine levels reached 52.79 mg/kg at the end of fermentation, while histamine and cadaverine levels remained below 30 mg/kg throughout the manufacturing process. The fermentation process also produced 7.64 mg/kg of phenylethylamine.

**Figure 6:**
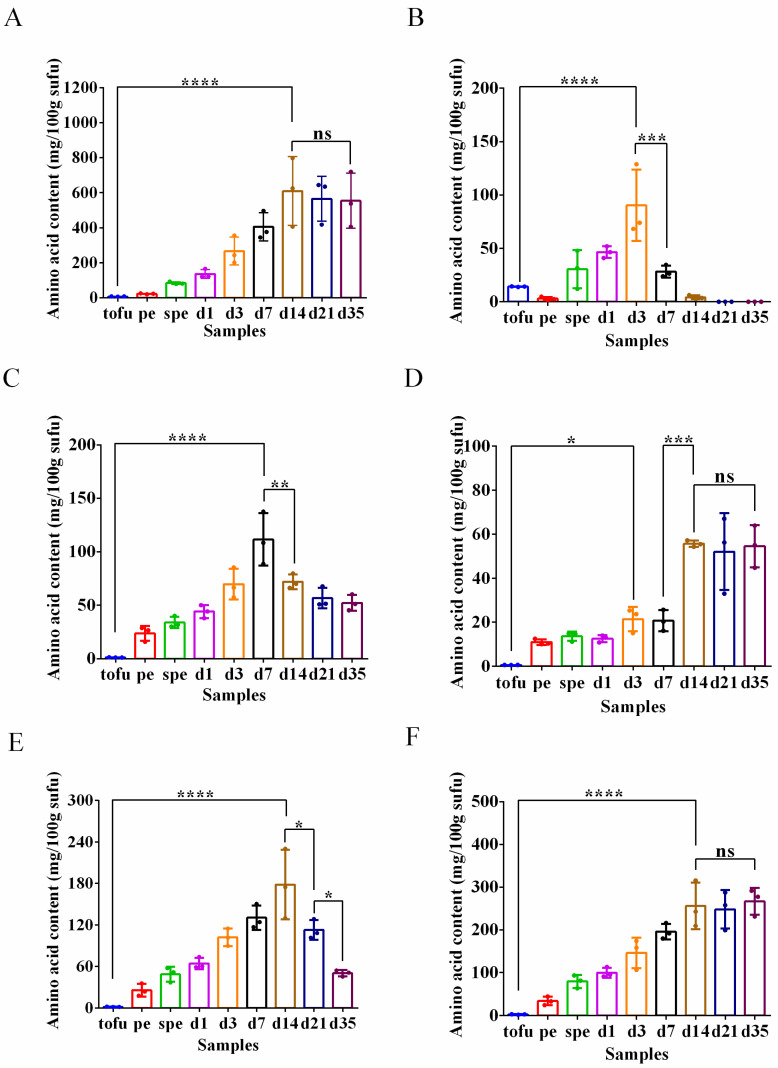
The alterations in glutamate (**A**), arginine (**B**), lysine (**C**), histidine (**D**), tyrosine (**E**), and phenylalanine (**F**) throughout the entire fermentation period of gray sufu. The data are presented as three replicates and the mean value. ns: no significance; *: *p* < 0.05; **: *p* < 0.01; ***: *p* < 0.001; ****: *p* < 0.0001. pe: pehtze; spe: salted pehtze; d1, d3, d7, d14, d21, and d35: the ripening time of sufu samples.

Microorganisms employ amino acid decarboxylases to transform precursor amino acids into BAs [36]. Histidine is the precursor of histamine [37], while glutamate and arginine are needed to produce putrescine [38,39]. The lysine, tyrosine, and phenylalanine are utilized to produce cadaverine, tyramine, and phenylethylamine [40,41], respectively. After 14 days of ripening fermentation, glutamate was discovered to be the most prevalent amino acid, with a concentration of 609.95 mg/100 g sufu (Figure 6A). Decarboxylating microorganisms used the accumulated amino acids to produce the BAs, causing the level of the six amino acids to increase and subsequently decline. Arginine levels declined immediately (7 days) (Figure 6B), followed by lysine (14 days) (Figure 6C), and the other four amino acids exhibited different degrees of decrease at 21 days (Figure 6A,D–F). Variability in the timing of the drops of the levels of different amino acids suggested that the peak synthesis of BAs was probably dynamic. The lower production of phenylethylamine can be explained by less reduction in phenylalanine level (Figure 6F). Furthermore, the degree of amino acid consumption demonstrated that tyramine and putrescine were the major BAs in these samples of gray sufu.

### 3.5. Correlations between Microbiota and Physicochemical Properties during Sufu Fermentation

**Figure 7:**
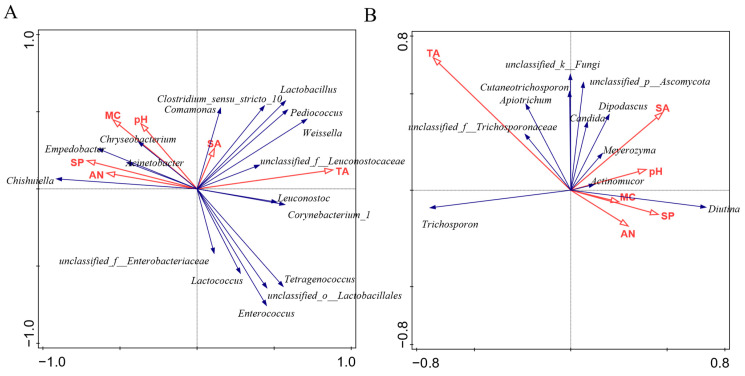
The redundancy analysis between bacterial (**A**), fungal (**B**) genera, and physicochemical properties was performed. Blue arrows indicate different microorganism genera, and red arrows indicate physicochemical properties. SA: salinity; TA: total acid; MC: moisture content; AN: amino nitrogen; SP: soluble protein.

To investigate the interplay between microorganisms and the physicochemical properties of gray sufu, a redundancy analysis was conducted using the abundant genera (OUT > 1%) in relation to specific physicochemical properties (Figure 7). As depicted in Figure 7A, positive correlations were observed between *Pediococcus*, *Weissella*, and *Lactobacillus* with respect to total acid content and salinity. *Lactococcus* and *Enterococcus* exhibited negative correlation coefficients with moisture content and pH, while *Chryseibacterium* and *Acinetobacter* displayed positive correlation coefficients with these properties. *Chishuiella* and *Empedobacter* showed positive associations with soluble protein and amino nitrogen and demonstrated negative correlations with total acid content. In Figure 7B, the relationships between dominant fungi and physicochemical properties are illustrated. *Diutina* displayed positive correlation coefficients with pH, moisture content, soluble protein, and amino nitrogen, while exhibiting negative correlation coefficients with total acid content. In contrast, *Trichospron* demonstrated positive correlation coefficients with total acid content and negative correlation coefficients with pH, moisture content, soluble protein, and amino nitrogen.

### 3.6. Correlations between Microbiota and Biogenic Amine during Sufu Fermentation

**Figure 8:**
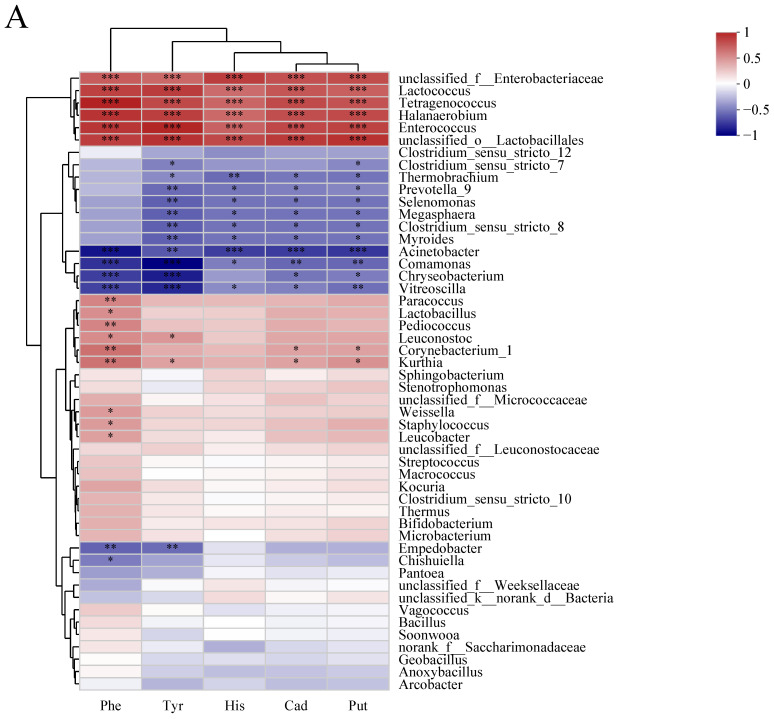

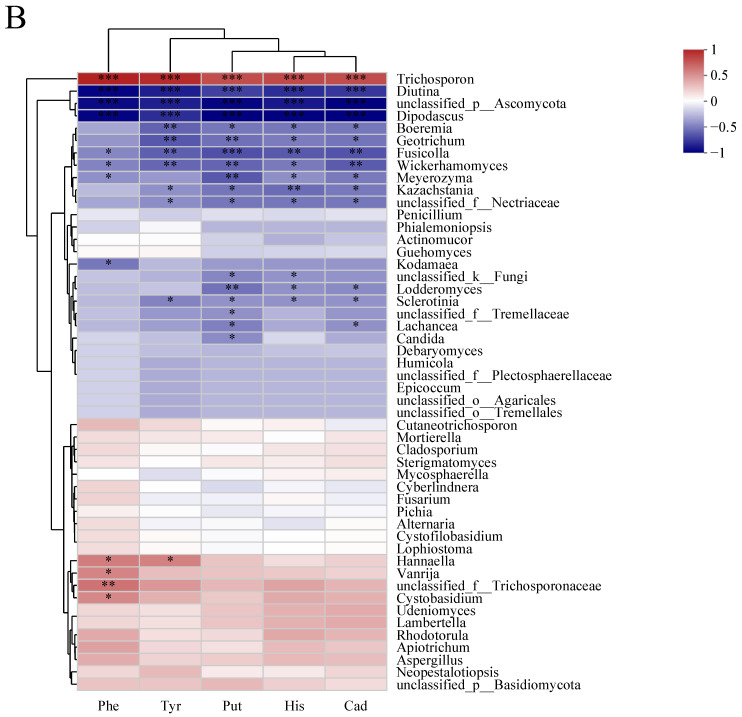
The correlation between microbial community and BAs in different fermentation times. (**A**) bacterial community and (**B**) fungal community. *: *p* < 0.05; **: *p* < 0.01; ***: *p* < 0.001.

Based on Spearman’s correlation coefficients and *p*-values, correlations between BAs and the 50 most abundant genera were examined. In this study, correlations between 28 bacterial genera and BAs were statistically significant (*p* < 0.05) (Figure 8A). Among these genera, *Lactococcus*, *Enterococcus*, *Tetragenococcus*, and *Halanaerobium* demonstrated significant positive correlations with each biogenic amine (*p* < 0.001), while *Acinetobacter* demonstrated substantial negative correlations with each biogenic amine (*p* < 0.01). As opposed to the other four BAs, *Paracoccus*, *Lactobacillus*, *Pediococcus*, *Weissella, Staphylococcus*, and *Leucobacter* were positively related to phenethylamine (*p* < 0.05). *Thermobrachium*, *Selenomonas*, *Megasphaera*, *Clostridium sensu stricto* 8, and *Myroides* were strongly negatively linked with the other four BAs, with the exception of phenylethylamine (*p* < 0.05). In terms of fungi, 22 genera were discovered to be significantly correlated with BAs (*p* < 0.05), with the majority exhibiting a negative connection (Figure 8B). *Trichosporon*, unlike *Diutina*, showed significant positive correlations with five BAs. In addition, the distribution of correlations between phenylethylamine and fungal communities differed significantly from that of the other four BAs.

## 4. Discussion

Gray sufu, a popular plant-based food product known for its distinctive flavor, is widely consumed in China and certain regions of Asia [42]. The production of gray sufu involves an open fermentation process, exposing the fermentation matrix to diverse environmental microorganisms (Figure 1). This fermentation environment leads to the formation of a spatially specific microbial community structure of gray sufu. Furthermore, the complex microbial structure and protein-rich nature of gray sufu create conditions conducive to biogenic amine (BA) production, which can accumulate during fermentation and pose risks to the safety and quality of the final product. This study focused on Shaoxing gray sufu, a widely consumed variety in eastern China, aiming to gain a comprehensive understanding of the relationship between microbial communities, physicochemical characteristics, and BA production.

In this study, the 16S rRNA gene sequences indicated that *Weissella*, rather than *Cyanobacteria* as reported by Liang et al. [43], was the predominant genus in tofu, aligning with the findings of Gu et al. [44]. As depicted in Figure 2A, the abundances of *Chishuiella*, *Acinetobacter*, and *Enterococcus* increased rapidly from tofu to pehtze phases. *Chishuiella*, known to be abundant in the brine of gray sufu, is a freshwater bacterium, while *Acinetobacter* represents widespread environmental bacterial contaminants [45]. Therefore, the rise in *Chishuiella* and *Acinetobacter* may be attributed to either the water or the environment. In contrast to *Chishuiella* and *Acinetobacter*, whose populations declined towards the end of fermentation, *Enterococcus* continued to expand as fermentation progressed (Figure 2). In this study, *Chishuiella*, *Enterococcus*, *Lactococcus*, and *Weissella* emerged as the dominant bacterial genera involved in gray sufu fermentation. It is worth noting that the variation in samples may account for the inconsistent findings compared to previous studies. In gray sufu products from Changchun province, *Lactobacillus*, *Bacillus*, *Pediococcus*, and *Pseudomonas* were the core bacterial genera. In fermented soybean products from Korea, *Tetragenococcus*, *Leuconostoc*, *Lactobacillus*, and *Enterococcus* were the predominant bacteria [46]. In doubanjiang-meju produced from broad beans in China, *Tetragenococcus*, *Acinetobacter*, *Pseudomonas*, and *Staphylococcus* were the prevailing genera during fermentation [47]. *Tetragenococcus*, known as halophilic lactic acid bacteria, typically dominates in fermented foods. However, in our study, *Tetragenococcus* did not appear to be a significant genus. Biological diversity in products can be greatly influenced by bacteria in the native environment. Regarding fungi, the abundance of *Diutina* and *Trichosporon* showed a fluctuating pattern, with *Trichosporon* constituting over 90% of the final product (Figure 3). Liang et al. reported that *Trichosporon* was most prevalent in raw tofu, while *Aspergillus* became the most abundant fungus in gray sufu products [48]. Thus, the variation in the microbial community may be attributed to differences in raw material sources, fermentation conditions, and procedures.

In fermented foods, pH and salinity play a crucial role not only in determining microbial structure but also in determining their quality and shelf life [49]. The pH value in tofu was the lowest (5.43) and significantly higher at the pehtze phase than at the tofu phase, as reported previously [50]. This pH increase is likely attributed to the enzymatic activity of microorganisms involved in the breakdown of tofu proteins into amino acids, followed by deamination processes that result in ammonia formation during the pehtze phase [51]. Additionally, changes in total acid content did not correspond to changes in pH during ripening fermentation, indicating that other factors may influence the pH of the samples (Figure 4A). Amino acid nitrogen and soluble protein are important nutrients present in gray sufu that can be readily absorbed, serving as indicators of the fermentation degree of gray sufu [5]. Proteolysis played a significant role as gray sufu aged with the dressing mixture, decomposing proteins into small molecular peptides and amino acids, subsequently increasing amino nitrogen content (Figure 4B) [52]. By the end of fermentation, the soluble protein content of the samples reached 6.35 g/100 g sufu, highlighting gray sufu as an excellent source of easily absorbed proteins (Figure 4B). Salinity peaked during the salted pehtze phase and significantly decreased after dressing mixture addition (*p* < 0.05) (Figure 4C). Due to similar osmotic pressures between sufu and mixture, salinity levels converged during ripening [5]. Moisture content showed a reciprocal relationship with salinity and remained stable after a period of ripening, consistent with previous studies [51]. Moreover, future studies should incorporate sensory analysis to evaluate attributes such as taste, texture, aroma, and appearance, providing a holistic assessment of gray sufu’s quality characteristics.

BAs are a group of small nitrogen-containing compounds produced through the decarboxylation of amino acids. Ingesting high levels of BAs can lead to abdominal cramps, headaches, and flushing [53]. This study investigated the changes in the content of five BAs during the fermentation of Shaoxing gray sufu (Figure 5). At the end of fermentation, the average levels of total BAs in gray sufu were 396.80 mg/kg, with phenylethylamine, histamine, cadaverine, putrescine, and tyramine averaging 7.64, 18.16, 20.43, 52.79, and 297.78 mg/kg, respectively. Due to their high toxicity, the European Food Safety Authority (EFSA) recommends that a single dose of histamine or tyramine not exceed 50 mg or 600 mg, respectively [54]. Brink et al. suggested that levels of histamine, tyramine, and phenylethylamine in food should be below 100, 800, and 30 mg/kg, respectively [55]. Using these recommendations as a benchmark, there was no probable exceedance in the samples collected for this study. However, considering the potential synergistic effects of various BAs [56], further research is required to establish maximum limits for combinations of different BAs.

Fermentation of protein-rich raw materials, such as soybeans, provides abundant precursor amino acids for BA synthesis [57]. The levels of six precursor amino acids were determined in order to gain insight into the process of synthesis of BAs during gray sufu fermentation (Figure 6). These amino acids were histidine, glutamate, arginine, lysine, tyrosine, and phenylalanine. The levels of these amino acids fluctuated, indicating their utilization in BA synthesis at different phases. Putrescine synthesis involves the decarboxylation of arginine by arginine decarboxylase (ADC) or the utilization of glutamate to synthesize N-acetylglutamate-5-phosphate, which is further converted to ornithine through ornithine decarboxylase (ODC) [58]. Consequently, glutamate was utilized for putrescine synthesis in the absence of arginine.

Redundancy analysis was conducted to examine the relationship between abundant microorganisms (OUT > 1%) and physicochemical factors (Figure 7). Among the bacterial genera analyzed, *Lactobacillus* demonstrated positive correlations with total acid content and salinity, while *Chishuiella* displayed positive associations with amino nitrogen and soluble protein. However, *Enterobacter* and *Leuconostoc* exhibited negative correlations with amino nitrogen, contrary to the findings reported by Liang et al. [43]. Similarly, *Enterococcus* showed opposing correlations with amino acid nitrogen in the studies conducted by Liang et al. [43] and Ding et al. [5]. Regarding the fungal genera, *Trichosporon* and *Diutina* were identified as the dominant genera. The RDA results indicated that *Trichosporon* exhibited positive correlation coefficients with total acid content but negative correlation coefficients with amino nitrogen, soluble protein, pH, moisture content, and salinity. On the other hand, *Diutina* displayed a positive correlation with pH, soluble protein, amino nitrogen, and moisture content while showing a negative correlation with total acid content. Previously, *Diutina* was not studied, and *Trichosporon* showed a negative correlation with moisture content and pH but a positive correlation with amino nitrogen [43]. Therefore, it is important to recognize that microbial communities are highly complex and dynamic, and their interactions with physicochemical factors can be intricate. The relationships between microorganisms and physicochemical properties are not always straightforward and can be influenced by various factors, such as the specific conditions of the fermentation process, the composition of the raw materials, and the presence of other microorganisms. Some of the observed differences may be the result of specific interactions or adaptations of certain bacterial genera in response to the unique conditions of each study. The underlying mechanisms behind the observed differences between certain bacterial genera and physicochemical factors require further investigation.

Furthermore, a Spearman correlation analysis was performed to investigate the relationships between BAs and the 50 most abundant genera of bacteria or fungi (Figure 8). The results indicated that bacteria exhibited significantly higher correlations with BAs compared to fungi, consistent with previous studies [59]. Among bacteria, 28 genera showed statistically significant correlations (*p* < 0.05) with BAs (Figure 8A). *Acinetobacter* exhibited significant negative correlations with all five BAs, while *Lactococcus*, *Enterococcus*, *Tetragenococcus*, and *Halanaerobium* showed significant positive correlations with these BAs (*p* < 0.001). Introduced from the environment, *Lactococcus* and *Tetragenococcus* increase the content of biogenic amines, which are decarboxylated to produce free amino acids [60]. The association pattern between phenylethylamine and bacterial communities was different from that of the other four BAs, which was similar to the findings of Ding et al. [5]. *Selenomonas, Myroides*, and *Megasphaera* were significantly negatively correlated with the other four BAs, except for phenylethylamine (*p* < 0.05). Similarly, significant associations were observed between 22 fungal genera and BAs, with most of them exhibiting negative correlations (Figure 8B). *Trichosporon* showed significant positive correlations with all five BAs, unlike *Diutina*.

In summary, our study provides a robust foundation for future research and practical applications in the field of gray sufu fermentation. Future investigations involving functional metagenomics, isolation of BA-producing and BA-degrading strains, exploration of microbial interactions, and sensory analyses will further advance our understanding of the complex microbial dynamics associated with sufu fermentation. This knowledge will enable the development of targeted strategies to mitigate biogenic amine production, enhance food safety, and improve the overall quality of gray sufu products. Ultimately, these endeavors will benefit both the scientific community and consumers by ensuring the production of high-quality and safe gray sufu products.

## 5. Conclusions

This study provided novel insights into the intricate microbiological structure of Shaoxing gray sufu fermentation. The predominant bacterial genera involved in sufu fermentation, including *Chishuiella*, *Enterococcus*, *Lactococcus*, and *Weissella*, were identified. Over the ripening process, the dominant fungus shifted from *Diutina* to *Trichosporon*. The BA content in gray sufu reached a peak of 396.80 mg/kg by the end of fermentation, with tyramine accounting for over 75% of the total BA content. Through redundancy analysis and Spearman correlation coefficients, it was demonstrated that the dominant microorganisms played a significant role in influencing the quality and safety of gray sufu. These microorganisms exhibited strong correlations with physicochemical parameters and biogenic amine production. Exploring and manipulating the microbial composition of gray sufu presents a promising strategy to enhance the overall quality of sufu. Future research endeavors will focus on isolating and identifying functional microorganisms that are relevant for improving the texture and sensory properties of gray sufu and promoting biogenic amine degradation. Comparative analyses of microbial community diversity in sufu raw materials, production processes, and products from different producers will be essential for the development of standardized fermented food safety guidelines.

## Data Availability

Data is contained within the article or Supplementary Material.

## Supplementary Materials

The following supporting information can be downloaded at: https://www.mdpi.com/article/10.3390/foods12142767/s1. Table S1: The condition of information on ion pairs, collision energies, and elution times for each biogenic amine; Table S2: The condition of amino acid analyzer.
